# Cardiac Xenotransplantation: A Narrative Review

**DOI:** 10.31083/j.rcm2507271

**Published:** 2024-07-24

**Authors:** Phan Quang Thuan, Nguyen Hoang Dinh

**Affiliations:** ^1^Department of Adult Cardiovascular Surgery, University Medical Center HCMC, University of Medicine and Pharmacy at Ho Chi Minh City, 72714 Ho Chi Minh City, Vietnam; ^2^Department of Cardiovascular and Thoracic Surgery, Faculty of Medicine, University of Medicine and Pharmacy at Ho Chi Minh City, 72714 Ho Chi Minh City, Vietnam

**Keywords:** artificial intelligence (AI), cardiac xenotransplantation (cXT), genetic modifications (GM)

## Abstract

Cardiac xenotransplantation (cXT) has emerged as a solution to heart donor scarcity, prompting an exploration of its scientific, ethical, and regulatory facets. The review begins with genetic modifications enhancing pig hearts for human transplantation, navigating through immunological challenges, rejection mechanisms, and immune responses. Key areas include preclinical milestones, complement cascade roles, and genetic engineering to address hyperacute rejection. Physiological counterbalance systems, like human thrombomodulin and endothelial protein C receptor upregulation in porcine xenografts, highlight efforts for graft survival enhancement. Evaluating pig and baboon donors and challenges with non-human primates illuminates complexities in donor species selection. Ethical considerations, encompassing animal rights, welfare, and zoonotic disease risks, are critically examined in the cXT context. The review delves into immune control mechanisms with aggressive immunosuppression and clustered regularly interspaced palindromic repeats associated protein 9 (CRISPR/Cas9) technology, elucidating hyperacute rejection, complement activation, and antibody-mediated rejection intricacies. CRISPR/Cas9’s role in creating pig endothelial cells expressing human inhibitor molecules is explored for rejection mitigation. Ethical and regulatory aspects emphasize the role of committees and international guidelines. A forward-looking perspective envisions precision medical genetics, artificial intelligence, and individualized heart cultivation within pigs as transformative elements in cXT’s future is also explored. This comprehensive analysis offers insights for researchers, clinicians, and policymakers, addressing the current state, and future prospects of cXT.

## 1. Introduction 

Cutting-edge medical interventions for advanced heart failure have demonstrated remarkable efficacy [1, 2, 3]. Nevertheless, after exploring all alternative treatment options, heart transplantation (HTx) persists as the preferred approach for patients in the advanced stages of heart disease, offering a substantial probability of prolonged and healthy life. Regrettably, the limited supply of human organs for transplantation has led to extensive waiting lists, with the annual demand far exceeding the actual number of transplants conducted [4]. In the United States, approximately 300,000 individuals are grappling with advanced heart failure. Despite a historic peak of 3817 patients undergoing allogeneic orthotopic HTx in 2021, the transplant waitlist still harbors over 3400 patients [5].

In the pursuit of alternative solutions, researchers are contemplating more daring approaches to donor selection, like considering organs from hepatitis C-positive brain-dead individuals [4, 6]. Another approach currently under scrutiny is donation following circulatory death [6, 7].

Presently, mechanical support devices serve as a crucial alternative to HTx; however, these devices are associated with elevated complication rates and offer only modest enhancements in patients’ quality of life. The survival rates at 1 and 5 years post-implantation for individuals reliant on these devices are 83% and 52%, respectively, marking a significant increase in morbidity compared to allogeneic heart transplantation [8]. Following the implantation of assistive devices, there is a noteworthy surge in hospital readmission rates, primarily attributed to complications such as infection and bleeding. Notably, 36% and 68% of readmissions occur at 3 and 12 months after surgery, respectively. The primary cause of mortality in these cases is the discontinuation of care [8].

Hence, the imperative quest for an alternative organ source, distinct from conventional organ donation, emerges as a pressing concern within the realm of medical healthcare. Recent investigations into cardiac xenotransplantation (cXT), involving the transplantation of pig hearts into humans, have yielded encouraging preliminary outcomes [4, 9]. This article provides a comprehensive review of human cardiac xenotransplantation, synthesizing existing evidence to offer insights into the current state of affairs while also contemplating future perspectives.

## 2. Cardiac Xenotransplantation

The inaugural cardiac transplantation occurred through a xenotransplantation procedure wherein a chimpanzee heart was transplanted into a 68-year-old male afflicted with an extensive history of hypertensive cardiovascular disease. This groundbreaking event, orchestrated by Dr. Hardy, occured on January 23, 1964 [10, 11]. Due to the inadequate size of the chimpanzee heart to accommodate substantial venous return and sustain proper circulation, this pioneering procedure lasted for 90 minutes.

Alongside the endeavors of cardiac surgeons in the 1960s, this period witnessed notable advancements in ABO compatibility, human leukocyte antigen (HLA) typing, and immunosuppressive agents [12, 13].

Significantly, progress in comprehending ABO compatibility and cross-matching resulted in enhanced early clinical outcomes in renal transplantation. The pioneering identification of the first-generation calcineurin inhibitor, cyclosporine, reshaped the landscape of cardiac transplantation. By 1982, it became evident that combining cyclosporine with low-dose steroids exceeded the efficacy of azathioprine and high-dose steroids, decreasing both early rejection and infectious complications in transplanted hearts [14].

Building upon medical advancements in the 1960s and 1970s, the inaugural neonatal cXT occurred on October 26, 1984, involving the transplantation of a baboon heart into an infant with hypoplastic left heart syndrome (HLHS) [15]. The infant, famously referred to as “Baby Fae”, possessed a rare O, Rh+ blood type among baboons. Comprehensive pretransplant immunologic assessments, encompassing lymphocytotoxic crossmatching and mixed lymphocyte cultures, suggested serum compatibility and anticipated limited responsiveness to xenogeneic lymphocytes. Despite enduring for 20 days, Baby Fae ultimately succumbed to humoral factors that resisted cyclosporine-based immunosuppression. Subsequent examination postmortem unveiled microvascular occlusions and interstitial hemorrhage, corroborating the involvement of humoral rejection (refer to Table 1, Ref. [6, 10, 11, 15, 16, 17, 18, 19, 20, 21, 22, 23, 24, 25, 26, 27, 28, 29, 30, 31, 32, 33, 34, 35]) [14]. One year following the “Baby Fae” case, another momentous heart allotransplantation took place. The recipient, a male infant diagnosed with HLHS, underwent orthotopic heart allotransplantation on November 20, 1985, receiving the heart from a brain-dead infant compatible in ABO blood type. Comprehensive histocompatibility testing revealed compatible lymphocytotoxic crossmatching, and subsequent immunosuppression involved cyclosporine, azathioprine, and prednisone. Dubbed “Baby Moses”, the patient has thrived into adulthood and remains in good health to this day [14].

**Table 1.** **History of Cardiac Xenotransplantation**.

Year	Summary	Reference
1964	Initial cardiac xenotransplantation (cXT) (chimpanzee-to-human)	[6, 10, 11]
1964	Human leukocyte antigen (HLA) typing technology	[16]
1967	T-cell depletion via anti-thymocyte globulin	[17]
1968	cXT (Pig-to-human)	[18]
1968	cXT (sheep-to-human)	[19]
1976	Discovery of cyclosporine	[20, 21]
1984	First neonatal cXT (baboon-to-human; “Baby Fae”)	[15]
1987	Identification of tacrolimus	[22]
1991	In-depth exploration of the mechanisms underlying rejection in cross-species transplantation	[23]
1995	Initial genetic engineering efforts aimed at addressing hyperacute rejection	[24]
1997	First orthotopic cXT using transgenic human CD59 expressing xenograft (pig-to-baboon)	[25]
2003	Manufacture of pigs lacking α1,3-galactosyltransferase	[26]
2005	The inaugural HTx in baboons employing alpha1,3-galactosyltransferase gene-knockout pigs as donors	[27]
2012	A new monoclonal antibody targeting CD40 extends the survival of islet allografts	[28]
2015	Heterotopic cXT employing TKO pigs featuring transgenic expression of human CD46 and human thrombomodulin, in combination with anti-CD40 antibody (pig-to-baboon)	[29]
2017	Generation of pigs with inactivated porcine endogenous retroviruses	[30]
2018	Implementing non-ischemic continuous preservation of porcine xenografts is proposed as a strategy to mitigate perioperative cardiac xenograft dysfunction in orthotopic heart xenotransplantation (pig-to-baboon). This approach has demonstrated success, enabling graft survival for up to 195 days	[31]
2021	Implementing the growth hormone receptor knockout serves as a preventive measure to curb post-transplantation xenograft overgrowth in pig-to-baboon xenotransplantation	[32]
2022	Progressive GM of porcine cardiac xenografts extend survival to 9 months (pig-to-baboon)	[33]
2022	The pioneering porcine-to-human cardiac xenotransplantation utilizing a source with ten gene modifications	[34]
2023	In 2023, a heart transplant from a pig to a 58-year-old patient with severe vascular issues was conducted. Unfortunately, the patient passed away 40 days later, with rejection being a possible cause (Baltimore group)	[35]

GM, genetic modifications; TKO, triple-knock-out; CD, cluster of differentiation; HTx, heart transplantation.

This raises a pivotal inquiry surrounding the disparities in genotypes between species that have impeded the initial outcomes of cXT from attaining the anticipated success. Subsequently, the progression of cardiac transplantation, grounded in a profound understanding of genetics and propelled by genetically modified trials involving pig-to-baboon, has gradually yielded positive results. Notably, a significant milestone was reached in January 2022 when the University of Maryland Medical Center, Baltimore, United States of America (USA), conducted the inaugural compassionate use of xenotransplantation involving a genetic modifications (GM) pig heart transplanted into a patient with terminal heart failure (see Table 1) [34, 36]. Although the patient’s passing after two months due to various complications is unfortunate, this achievement represents a significant step forward in demonstrating the feasibility of clinical cXT. The sustained normal heart function for over 45 days underscores the potential of this approach in addressing critical cardiac conditions [6]. The Baltimore group performed a second pig-to-human heart transplant on September 20, 2023, for a 58-year-old patient deemed unsuitable for allogeneic heart transplantation due to severe peripheral vascular disease and internal bleeding complications. Regrettably, the patient passed away 40 days after the transplant, likely due to early signs of rejection [4].

## 3. Selection of Animal Heart Donors

Pigs are considered optimal for organ donation in human cXT due to their ease of breeding, rapid maturation, attainment of adult human size within months, and cardiac anatomy closely resembling that of humans in both size and function (Table 2, Ref. [4, 9, 14, 34, 37]) [9, 38, 39, 40, 41].

**Table 2.** **Comparative Analysis of Pigs and Baboons as Heart Donors**.

	Pig	Baboon	Reference
Physiological Similarities	Moderately close	Close	[9, 37]
Resemblance in anatomy to humans	Moderately close	Close	
Availability	Unlimited	Limited	
Growth	Rapid (6 months)	Slow (9 years)	
Adult organ dimensions	Adequate	Inadequate	
Period to reproductive maturity	4–8 months	3–5 years	
Length of pregnancy	114 ± 2 days	173 ± 193 days	
The quantity of offspring	5–12	1–2	
Cost of maintenance	Significantly lower	High	
The immune system’s correlation with humans	Distant	Close	
Understanding tissue compatibility	Considerable (in selected herds)	Limited	
Expertise in genetic manipulation	Considerable	None	
Potential for infection transmission (xenozoonosis)	Low	High	
Requirement for compatibility of blood types with humans	Probably unimportant	Important	
Availability of specific pathogen-free animals	Yes	No	
Public opinion	More in favor	Mixed	
Xenotransplantation research history	Research experience	Limited	[4, 14, 34]

While pigs are considered an ideal species for organ harvesting due to their rapid growth to human size within a few months, the genetic engineering of pigs has advanced significantly. Pigs now possess hearts that are structurally and functionally similar to the human heart. However, the use of pigs as a source of organs has raised ethical concerns, particularly from animal rights activists who emphasize the intelligence, sentience, and capacity for suffering of these animals [42, 43].

Opponents of using pigs for xenotransplantation question whether it is ethically acceptable to exploit animals in this way, considering their historical role as a source of food. Additionally, ethical considerations arise from religious perspectives, with some traditions, such as Judaism and Islam, prohibiting the consumption of pork products while accepting porcine organ transplantation as a means to preserve human life [9, 44, 45]. Comparatively, baboons, being nonhuman primates with sophisticated social behaviors, present additional ethical challenges in their usage. In contrast, pigs are generally less contentious in this regard. Despite these ethical considerations, the analysis underscores that pigs remain potential candidates for cXT [9].

Due to their rapid reproductive and developmental cycles, pigs offer an extended timeframe for testing genetic modifications—a process that would be considerably time-consuming if conducted on alternative species, such as baboons (see Table 2). Additionally, their prolific offspring production facilitates comprehensive genetic observations, aiding in the selection of desired purebred breeds for specific GM [46]. Meanwhile, nonhuman primates (NHPs) demand extensive breeding facilities and substantial resources. Their extended time to maturity, slow reproductive rates, and tendency to yield undersized organs pose additional challenges. Moreover, the ethical and moral complexities associated with capturing and breeding NHPs for organ harvesting are considerable. Additionally, the potential for virus transmission between closely related species is a significant concern [5].

## 4. Genetic Modifications and Rejection

### 4.1 Hyperacute Rejection

Heart transplant rejection ensues when the recipient’s immune system reacts to foreign antigens present in the donor organ, triggering an immune response. Patients commonly exhibit symptoms of heart failure such as breathlessness, orthopnea, nocturnal dyspnea, palpitations, syncope, and related manifestations. Hyperacute rejection denotes the rapid identification of the transplanted organ by pre-existing antibodies targeting donor cells. This immediate reaction leads to endothelial damage and graft destruction within a matter of hours [5, 47].

During infancy, both humans and NHPs produce antibodies that target carbohydrate antigens found on unmodified pig cells. Consequently, when a normal pig organ is transplanted into a human or baboon, these antibodies quickly bind to the vascular endothelial cells of the graft. This triggers the activation of the complement cascade and attracts leukocytes, which infiltrate the porcine heart through various mechanisms, ultimately leading to graft rejection within minutes to hours. Known as “hyperacute rejection”, this rapid immune response, driven by antibodies, is characterized by histopathological features such as venous thrombosis, loss of vascular integrity, interstitial hemorrhage, edema, and infiltration of innate immune cells [4, 48].

Hyperacute and subsequent acute rejections of pig organs in humans or NHPs primarily arise from preexisting antibodies that target galactose-α-(1,3)-galactose (αGal). These preexisting antibodies resemble those formed against the A and B antigens that determine our blood type [5, 40, 46]. Humans possess inherent antibodies against N-glycolylneuraminic acid (Neu5Gc) and a glycan resembling the human Sd(a) blood group antigen (known as β4Gal). In contrast, NHPs only display anti-αGal and anti-Sd(a) antibodies [14, 49]. To negate αGal, Neu5Gc, and Sd(a) epitopes as targets for xenograft rejection in humans, pigs with inactivated α-1,3-galactosyltransferase (GGTA1), cytidine monophosphate-N-acetylneuraminic acid hydroxylase (CMAH), and β-1,4-N-acetyl-galactosaminyl transferase 2 (B4GALNT2)/B4GALNT2-like (B4GALNT2L) genes were developed, resulting in what is commonly known as “triple-knock-out (TKO) pigs” (see Table 3, Ref. [6, 14, 26, 29, 50, 51, 52, 53, 54, 55]) [4, 34].

**Table 3.** **Rejection and Genetic Modification**.

Obstacles	Genetic modifications	Rationale	Reference
Hyperacute rejection	Knockout of α-1,3-galactosyltransferase	Galactose-α-(1,3)-galactose (αGal) as the primary xenoantigen inducing hyperacute rejection in pig-to-human/primate xenotransplantation	[6, 26]
	(GGTA1-KO)		
	Knockout of cytidine monophosphate-N-acetylneuraminic acid hydroxylase	The removal of CMAH, the enzyme responsible for Neu5Gc synthesis, through knockout effectively eliminates the primary non-αGal xenoreactive antigen. This prevents the triggering of an innate immune response in humans	[6, 53]
	(CMAH-KO)		
	Knockout of β-1,4-N-acetyl-galactosaminyl transferase 2 (B4GALNT2-KO)	Eliminates the glycan resembling human Sd(a), for which humans/primates develop preformed antibodies	[6, 54]
	Expression of human CD46, CD55, CD59	CD46, CD55, CD59 serve as complement regulatory proteins (CRPs), downregulating complement activation. Expressing it helps to suppress complement activation	[50, 51, 52]
	Expression of human thrombomodulin	Human TBM functions as an anticoagulant protein, essential for overcoming coagulation incompatibilities following pig-to-primate/human xenotransplantations	[6, 55]
	(TBM)		
	Expression of human endothelial protein C receptor (EPCR)	Human EPCR is an anticoagulant protein that facilitates the formation of the TBM-thrombin complex	[6, 29]
Antibody mediated rejection	Inhibition of CD40-CD40L co-stimulation through the use of the chimeric 2C10R4 anti-CD40 monoclonal antibody	CD40 is expressed on B cells, and CD4+ helper T cells express CD40L. This interaction leads to the activation of B cells and the generation of humoral antibodies against the processed antigen	[14]
Cellular rejection	Transgenic expression of human CD47	Physiologically, macrophage activation is regulated by the inhibitory interaction between signal-regulatory protein alpha (SIRPα) and CD47, known as the ‘do not eat me signal’. The absence of CD47 on porcine endothelial cells has the potential to induce macrophage activation	[14]

Neu5Gc, N-Glycolylneuraminic acid; CD, cluster of differentiation.

Another significant aspect of hyperacute rejection pertains to complement activation. Under normal physiological circumstances, complement proteins circulate in the bloodstream, serving a crucial role in identifying and neutralizing blood-borne pathogens. As a protective measure, humans produce complement regulatory proteins (CRPs) to prevent inadvertent complement activation at the interface between organs and blood. However, pig CRPs are ineffective at completely inhibiting human complement proteins, resulting in excessive activation of the complement cascade, thus contributing to hyperacute rejection [5]. Complement activation can occur independently of antibody binding, triggered by pathways such as ischemia-reperfusion injury. To mitigate this risk, genetic engineering has been utilized to incorporate extra human complement pathway regulatory proteins (CPRPs), notably cluster of differentiation (CD)46, CD55, and CD59, into pigs [50, 51, 52]. Organs sourced from animals expressing transgenic human CPRPs display significant protection against complement-mediated injury in humans or NHPs. When combined with TKO pigs, these “humanized” porcine organs show a notable decrease in cellular damage [4].

Advanced genetic engineering techniques were utilized to inhibit the activation of porcine endothelial cells, alongside the complement cascade. In typical physiological circumstances, endothelial cell injury prompts the release of heparan sulfate. Human thrombomodulin (TBM) functions to prevent *in vivo* thrombus formation by activating the anticoagulant protein C [56]. Transgenic expression of TBM and endothelial protein C receptor (EPCR) was pursued to emulate the natural counterbalance system present in endothelial cells. This strategy led to a notable augmentation in protein C activation, decreased graft thrombosis, and an extended xenograft survival period [5, 14].

### 4.2 Antibody-Mediated Rejection and Cellular Rejection

Cellular and antibody-mediated rejection usually manifests weeks to months post-transplantation. The human immune system discerns “self” from “foreign” molecules via cell surface proteins encoded by major histocompatibility complex (MHC) genes, also termed HLA. In instances of HLA mismatches between donor and recipient, the recipient’s immune system may identify the donor organ as foreign, triggering an immune response and potentially leading to organ rejection [5].

To manage immune responses against pig antigens, aggressive immunosuppression therapy is necessary, involving thymoglobulin for T cell depletion, rituximab to suppress B-cell antibody production, and anti-CD40 antibodies to block immune cell co-stimulation. Xenoimplantation requires CD40-CD40L pathway blockade, leading to impaired B cell activation, affecting xenoantigens, immunoglobulin class switching, and germinal center reactions. This blockade is achieved using anti-CD40 monoclonal 2C10R4 antibodies, alongside mycophenolate mofetil (MMF) depletion of B and T cells, and complement depletion to mitigate antibody-mediated rejection [14, 34]. Additionally, natural killer (NK) cells and macrophages play pivotal roles in xenograft rejection. NK cells typically target virally infected or tumor cells by recognizing down-regulated MHC molecules. Macrophages contribute to rejection through the loss of inhibitory interaction between signal regulatory protein alpha (SIRPa) on macrophages and CD47 on porcine cells. CRISPR/Cas9 gene-editing technology has been employed to create pig endothelial cells expressing human inhibitor molecules like MHC I, CD33-related Siglecs, CD47, and CD200, aiming to diminish NK cell and macrophage activation [5, 39, 57].

## 5. Clinical Cardiac Xenotransplantation 

### 5.1 Patient Selection

The initial patient selection for a clinical trial of cardiac xenotransplantation demands meticulous evaluation to weigh inherent risks and ensure favorable outcomes. Suitable candidates may include individuals in intensive care units ineligible for mechanical circulatory support, such as those with hypertrophic obstructive cardiomyopathy, prior mechanical valve replacement, and post-infarction ventricular septal defect [4, 40, 58]. In these high-risk patients, instability often escalates due to their reliance on inotropes and the occurrence of arrhythmias. Assessing the potential reversibility of secondary liver and kidney damage, as well as the feasibility of addressing pulmonary hypertension, becomes imperative in such cases [46]. Neonates and infants afflicted with intricate congenital heart disease may find substantial advantages in cXT due to donor shortages and the difficulties along with less-than-ideal outcomes linked to mechanical circulatory support in this demographic [4, 59].

In 2000, the Advisory Committee to the International Society for Heart and Lung Transplantation proposed that consistent survival of NHPs supported by orthotopic porcine heart transplants for a duration of 3 months would be sufficient to justify a clinical trial [40, 60]. To date, no NHPs have exceeded a survival period longer than 9 months after being sustained by an orthotopic pig heart transplant. Consequently, regulatory bodies such as the US Food and Drug Administration (FDA) or European Medicines Agency (EMA) might suggest that cXT initially serves as a bridge, spanning several months [40]. During this period, a cardiac allotransplantation could subsequently be performed if deemed appropriate upon analysis. Moreover, adherence to patient consent, ethical guidelines established by international organizations, and compliance with the laws of the government in which the patient is selected are imperative [43]. Hence, in the current period, the selection of patients for cXT demands meticulous attention to ensure alignment with both professional and ethical considerations, in accordance with prevailing guidelines.

### 5.2 Clinical Case Pig to Human 

The groundbreaking cXT case, involving the world’s first genetically modified pig-to-human transplantation, occurred in 2022 at the University of Maryland Medical Center in the USA. The patient, a 57-year-old man with chronic mild thrombocytopenia, hypertension, nonischemic cardiomyopathy, and a history of mitral valve repair, was admitted due to severe heart failure, with a left ventricular ejection fraction of 10%. His treatment escalated, including multiple intravenous inotropic agents, and an intra-aortic balloon pump was inserted on hospital day 11. Subsequently, the patient faced ventricular arrhythmias leading to cardiac arrests, necessitating resuscitation. Peripheral venoarterial extracorporeal membrane oxygenation was initiated on hospital day 23 [34].

This patient, ineligible for allotransplantation and mechanical circulatory support due to poor treatment adherence, demonstrated preserved kidney function, meeting the specific criteria for cardiac xenotransplantation. The careful selection process involved thorough consultation with the patient, obtaining informed consent, and securing FDA approval [34]. Notably, this case served as a model for the second case at the University of Maryland Medical Center, emphasizing the significance of adherence to selection criteria in the evolving field of cXT [4].

The pig hearts utilized in xenotransplantation were derived from pigs genetically edited with 10 specific genes. This genetic modification involved the knockdown of four pig genes to eliminate major porcine xenoantigens and the inclusion of growth hormone receptor (GHR) to prevent xenograft overgrowth. Additionally, six human genes were introduced to regulate complement flow, modulate inflammatory responses, and prevent abnormal blood clotting [42]. The immunosuppressive regimen comprised rituximab and anti-thymocyte globulin (ATG) for B and T cell depletion, along with C1 esterase inhibitors to control the complement pathway. The primary strategy involved the use of an anti-CD40 monoclonal antibody (KPL-404) to block the CD40-CD40L co-stimulatory pathway, complemented by methylprednisolone pulses. Maintenance immuno-suppression included mycophenolate mofetil, KPL-404, and a tapered methylprednisolone regimen [14, 38]. At the 45-day post-transplantation mark, the patient exhibited no signs of rejection, and the transplanted heart functioned well. However, the patient’s condition later deteriorated, leading to death on the 60th day [4, 34]. While the specific cause of death remained uncertain, the recorded data suggested a promising future for xenotransplantation. Despite the challenges faced in this case, the outlook for the continued development of xenotransplantation appeared optimistic.

### 5.3 Recently Clinical Trial

In March 2024, a search on clinicaltrial.gov for interventional clinical trials related to xenotransplantation did not yield any results specifically for cXT. Although there are numerous clinical trials related to xenotransplantation in organs such as bone, skin, kidney, and liver [41], the absence of cXT trials suggests a cautious approach within the scientific community. However, it is conceivable that as scientific advancements progress in the near future, clinical trials specifically addressing cardiac xenotransplantation may emerge.

## 6. Ethical and Regulatory Aspects

Xenotransplantation is fraught with numerous ethical and social dilemmas, encompassing substantial costs and resource allocations essential for the research endeavor. Questions abound regarding the potential benefits of this scientific pursuit, along with lingering concerns regarding animal rights, animal welfare, and the genetic manipulation of animals designed for human consumption. Additionally, there is a pervasive apprehension surrounding the prospect of xenozoonosis, adding a layer of complexity to the ethical discourse surrounding this innovative field [6, 43, 61].

The advantages of cXT are evident, particularly in light of the extensive waiting list for donors coupled with the scarcity of donor organs [9, 62]. However, its true efficacy emerges when the long-term outcomes approach those of allotransplantation [6]. The application of GM techniques has been instrumental in bridging the gap between species, as demonstrated by the extension of survival time without rejection in humans with genetically modified pig hearts, reaching an impressive additional two months [35, 36]. These successes underscore the potential for cXT to evolve into a pivotal solution for the organ transplant shortage. Looking ahead, the remarkable progress in genetic technology, coupled with the support of artificial intelligence (AI), holds the promise of further elevating cardiac transplantation [63, 64, 65]. This trajectory suggests a future where the shortage of organs for transplant may be effectively addressed through the continued refinement of genetic techniques and the integration of advanced technologies.

Animal rights activists contend that animals possess emotions and resist being utilized as donors for heart transplants. Despite being a source of food for millennia, ethical concerns arise when considering the use of animals for living organs [66, 67]. Some religious traditions, such as Judaism and Islam, prohibit pork consumption, yet some leaders consider pig organ transplants acceptable to preserve human life. For vegetarians, an ethical dilemma arises regarding sacrificing the lives of animals to save others, since animals and humans are also sentient beings [68]. Despite these ethical considerations, pigs remain a suitable donor source for heart transplantation [42].

The interaction between a genetically modified pig heart and the human body, particularly concerning mental health, poses uncertainties. Recipient patients must be informed about this matter, considering its acceptance and addressing potential social issues [38, 69]. Currently, no cases of long-term societal reintegration following cXT exist for comprehensive monitoring. While this concern holds relevance, the immediate focus remains on developing a flawless pig heart capable of safe and permanent transplantation into the human body.

The potential for disease transmission from animals to humans is a critical concern in xenotransplantation. This has led to significant debates regarding mitigation strategies for both endemic and epidemic pathogens, exemplified by diseases like Human immunodeficiency virus infection and acquired immunodeficiency syndrome (HIV/AIDS), Ebola, avian flu (A/H5N1), and swine flu (A/H1N1). The irony is palpable when considering that the first pig heart transplant occurred during a pandemic. The devastating impact of COVID-19, caused by the zoonotic SARS-CoV-2 virus, highlights the risks associated with diseases transferring from animals to humans [42, 69]. However, advancements in clean livestock farming and gene technology offer promising avenues for effectively controlling and managing zoonotic challenges [35, 70, 71, 72].

## 7. Challenges and Future Directions

### 7.1 Immunological Control

The utilization of genetic modifications for immunological control marks a significant stride in cardiac xenotransplantation. The incorporation of CRISPR/Cas9 system technology in pig genetic modifications has yielded a pig heart capable of thwarting acute graft rejection upon transplantation into the human body—a miraculous advancement showcasing the remarkable progress in biotechnology within the realms of genetics and organ transplantation [4, 73]. Furthermore, the ongoing development of AI is poised to propel genetic biotechnology forward, paving the way for novel techniques to tailor animal donor genetics to align with the human immune system—an anticipated evolution. A prime illustration is AI’s contribution to predicting and optimizing genome editing methods like CRISPR/Cas9, a system instrumental in creating modified pigs for the inaugural pig-to-human cXT [63, 74, 75]. Notably, AI has the potential to expedite the exploration of new genetic technologies, bringing us closer to realizing the aspiration of a seamlessly accepted heart upon transplantation into the human body.

### 7.2 Zoonotic Infectious Disease Control

Porcine endogenous retroviruses are inherent in the genomes of all pigs, and designated pathogen-free breeding cannot entirely eliminate them. A critical apprehension regarding this group of viruses is their potential to infect human cells, undergo mutations leading to cancer, or amalgamate with other viruses, giving rise to novel infectious diseases. Recent breakthroughs, including the knockout of all proviruses in the pig genome through gene editing, mitigate these risks but do not reduce them to zero [42]. However, the continued progress in genetic technology and artificial intelligence holds the promise of achieving zero risk in the future [74].

Patients undergoing cardiac xenotransplantation may face the potential emergence of new viruses, precipitating an epidemic. Despite the current state-of-the-art genetic technologies reducing this risk to a minimum, the ability of patients to resume a normal life and reintegrate into society remains unpredictable [76]. Viruses like HIV, which originate from animals and are challenging to detect, necessitate vigilant monitoring of patients re-entering society to avert new outbreaks [42]. Patients may even need to exercise control over personal activities, such as sexual behavior, to minimize risks [38, 42]. In addition to genetic technology and AI contributing to diminishing the risk of zoonotic diseases in xenotransplantation, monitoring sensor technologies, coupled with AI, will play a pivotal role in closely tracking patients, ensuring the lowest possible risk of epidemic outbreaks following xenotransplantation [74]. While these concerns may not be immediate, the advancing landscape of xenotransplantation in the future will elevate them to crucial considerations for post-xenotransplantation patients and humanity at large.

### 7.3 Ethical and Social Considerations

As discussed in the section on Ethical and Regulatory Considerations, cXT presents numerous ethical challenges that must be effectively addressed to ensure the widespread acceptance of xenotransplantation. Consequently, the establishment of a committee dedicated to addressing xenotransplantation-related ethical concerns appears imperative. The Ethics Committee of the International Xenotransplantation Association assumes such a pivotal role [43, 77]. Since 2003, this organization has been instrumental in issuing guidelines on Ethics in Xenotransplantation, providing valuable guidance to ensure that xenotransplantation research adheres to ethical standards [45]. However, with ongoing scientific advancements, these ethical considerations are likely to evolve in the future, underscoring the significant responsibility and role of this organization as xenotransplantation becomes commonplace, and organ sources potentially become less constrained.

### 7.4 Future Directions

The objective of the cXT project is to make a heart in a pig’s body that closely resembles a human heart, demonstrating genetic traits that make it compatible when transplanted into a human recipient. Essentially, this genetically modified heart aims to mimic human cardiac characteristics, ensuring seamless integration into the human body without rejection. The realization of this goal hinges on advancements in genetic technology and AI, which are expected to play a crucial role in achieving this feat in the future [74, 78, 79].

In the future of Precision Medical Genetics, the aspiration is to grow an individual’s heart, encompassing its entire genome, within a pig. This groundbreaking approach seeks to obviate the requirement for immunosuppressive drugs in the recipient’s body. With advancing research on human genes and their regulatory functions, a prospective scenario foresees the storage of the genome of every human born [80, 81]. Utilizing genes that govern organ development, these organs can be engineered within animals, notably pigs. This methodology guarantees the utmost genetic uniformity, minimizing the risk of graft rejection, and stands as a trailblazing path for the future.

In the advanced and comprehensive landscape of xenotransplantation, the scarcity of heart donors will be eliminated, leading to an expansion of indications for heart transplantation. Xenotransplantation will no longer be confined to patients facing life-threatening diseases without access to conventional allotransplantation. Instead, it may emerge as a viable organ replacement option, extending beyond immediate life-saving needs. This marks the dawn of the era of regenerative medicine in cardiovascular medicine, paving the way for an extended human lifespan [82].

Furthermore, to anticipate the trajectory of the xenotransplantation era, it is essential to engage in research aimed at predicting and addressing ethical issues [9, 44]. This proactive approach seeks to prepare for potential scenarios and enhance patients’ comprehension of the effects of xenotransplantation on their post-transplant life, particularly for those who choose to participate in trials.

## 8. Standards in Implementing Cardiac Xenotransplantation

**Fig. 1.**
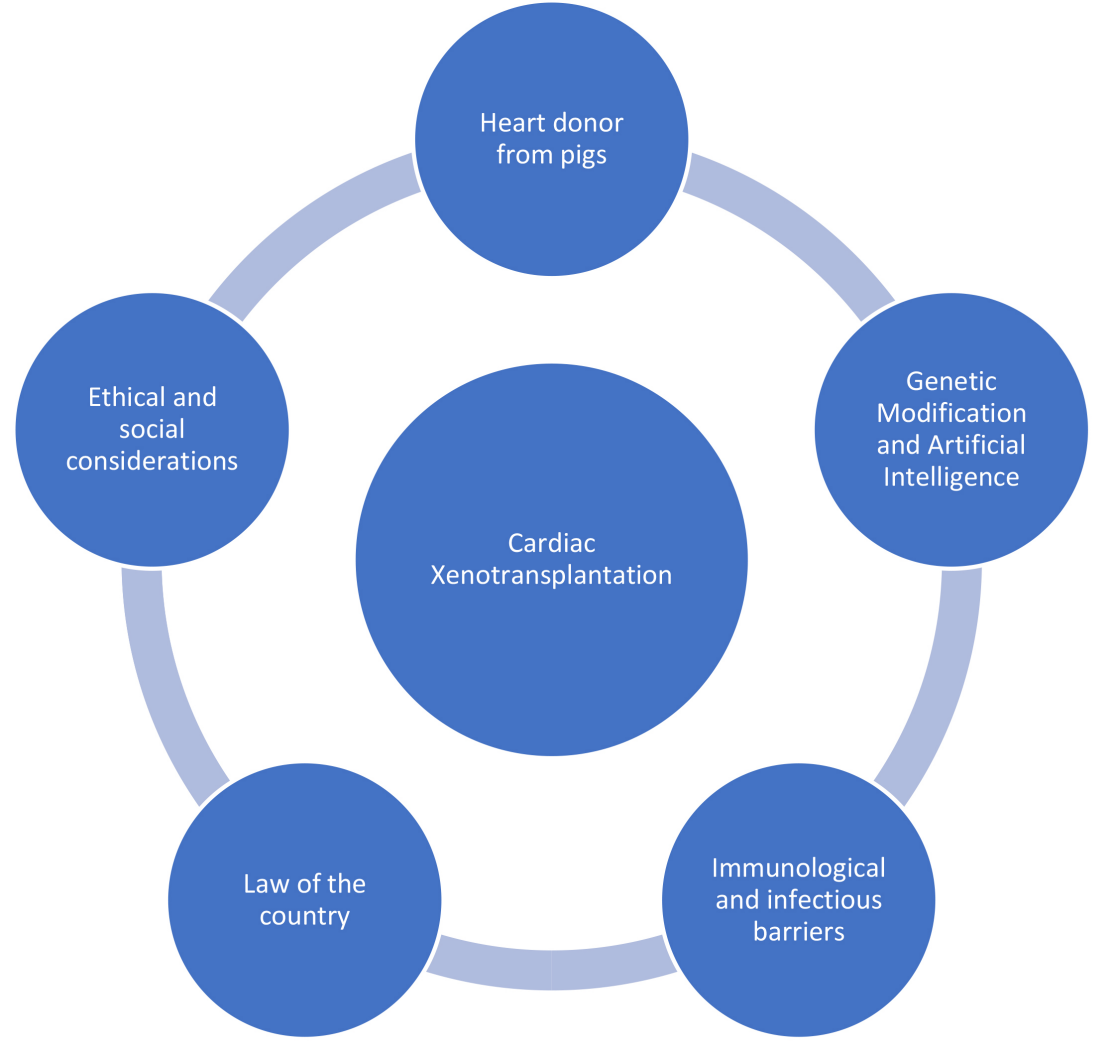
**Standards in Implementing Cardiac Xenotransplantation**.

Following the groundbreaking milestone of the inaugural gene-edited pig-to-human heart transplant in the USA, which entailed 10 distinct gene edits, subsequent successful cXT procedures have been performed at the University of Maryland School of Medicine [4, 34]. Despite the unfortunate outcome of the patients, who all succumbed within a few months post-transplant with an unknown rejection mechanism, these successes indicate a promising direction for research and the implementation of cardiac xenotransplantation. Subsequently, standards for implementing cXT have progressively emerged, providing a valuable reference for centers worldwide seeking to undertake such procedures (refer to Fig. 1) [5, 42, 83, 84].

In the selection of animals for heart transplantation research, as thoroughly reviewed in the previous sections, pigs have emerged as optimal candidates for experimental studies aimed at developing heart donors. While there are some differences in anatomical details, actual clinical cases reveal negligible echocardiographic disparities [83, 85]. Consequently, research centers in Germany, Korea, and China consistently choose pigs as the preferred animals for investigating and creating genetically modified heart donors [4, 14, 86]. Pigs are gradually solidifying their status as the standard in the field of genetically modified animals for heart donation.

The challenge in cardiac xenotransplantation lies in the human body’s ability to differentiate between “self” and “foreign”—the immune system’s inherent guide for determining what to eliminate and what to tolerate [83, 87]. Effective control of infectious and immunological rejection is crucial for the success of cardiac xenotransplantation. Advanced technologies, including modified genes and AI, are essential to make necessary adjustments tailored to the human body [88, 89]. The integration of technology with the support of AI will propel the success and further advancement of the cXT program in the future [74]. Countries adopting xenotransplantation are also actively pursuing these technologies to master the intricacies of cXT [4, 14, 86].

Addressing ethical considerations is crucial in allogeneic HTx, as interpretations of ethics may vary among countries based on societal norms. Prior to implementation, careful consideration of religious and other factors is essential to prevent unfavorable public opinion and secure consensus in clinical application [43, 83]. For instance, in the USA, the responsibility falls under federal laws, with the FDA overseeing. Germany, on the other hand, relies on the Ethics Committee of the International Xenotransplantation Association, following European Union guidelines and German law. In Korea, the ethical framework is established through the Biomedical Research Committee Ethics [4, 14]. Pursuing ethical and social issues is a common standard for cardiac xenotransplantation programs.

Each country operates under its unique legal system, which can impact the cardiac xenotransplantation process [40]. An illustrative example is seen in heart transplantation donors, where changing the legal policy from opt-in to opt-out for organ donation has significantly expanded the pool of donated organs [90, 91]. Therefore, before implementing cardiac transplantation, it is essential to establish a consensus on legal policies with health management agencies. For instance, the first case of cardiac xenotransplantation from pig gene modification received FDA approval and supervision [34]. Similarly, countries like Germany, Korea, and China are actively developing policies to advance this field [4, 14, 86]. Ensuring national legality becomes a standard in implementing cardiac heart transplantation.

## 9. Conclusions 

A significant population of individuals suffering from heart disease qualify as candidates for heart transplantation. However, the heart, being the organ with the most limited donor source in the body, can only be donated when the donor is no longer alive in the case of allotransplantation. Consequently, cardiac xenotransplantation emerges as a promising direction to address the shortage of transplanted organs. The continuous progress in genetic technology, artificial intelligence, and thorough ethical and legal considerations have propelled xenotransplantation toward numerous successes, setting standards for future clinical applications. Adhering to these standards, xenotransplantation is poised to achieve remarkable success in the future, offering enhanced life opportunities for patients.
